# Sample size recalculation in three-stage clinical trials and its evaluation

**DOI:** 10.1186/s12874-024-02337-9

**Published:** 2024-09-25

**Authors:** Björn Bokelmann, Geraldine Rauch, Jan Meis, Meinhard Kieser, Carolin Herrmann

**Affiliations:** 1 Charité - Universitätsmedizin Berlin, corporate member of Freie Universität Berlin and Humboldt-Universität zu Berlin, Institute of Biometry and Clinical Epidemiology, Charitéplatz 1, Berlin, 10117 Germany; 2 Institute of Medical Biometry, University Medical Center Ruprechts-Karls University Heidelberg, Im Neuenheimer Feld 130.3, Heidelberg, 69120 Germany; 3 Technische Universität Berlin, Straße des 17. Juni 135, Berlin, 10623 Germany

**Keywords:** Clinical trials, Adaptive trial design, Sample size adaptation, Performance evaluation

## Abstract

**Background:**

In clinical trials, the determination of an adequate sample size is a challenging task, mainly due to the uncertainty about the value of the effect size and nuisance parameters. One method to deal with this uncertainty is a sample size recalculation. Thereby, an interim analysis is performed based on which the sample size for the remaining trial is adapted. With few exceptions, previous literature has only examined the potential of recalculation in two-stage trials.

**Methods:**

In our research, we address sample size recalculation in three-stage trials, i.e. trials with two pre-planned interim analyses. We show how recalculation rules from two-stage trials can be modified to be applicable to three-stage trials. We also illustrate how a performance measure, recently suggested for two-stage trial recalculation (the conditional performance score) can be applied to evaluate recalculation rules in three-stage trials, and we describe performance evaluation in those trials from the global point of view. To assess the potential of recalculation in three-stage trials, we compare, in a simulation study, two-stage group sequential designs with three-stage group sequential designs as well as multiple three-stage designs with recalculation.

**Results:**

While we observe a notable favorable effect in terms of power and expected sample size by using three-stage designs compared to two-stage designs, the benefits of recalculation rules appear less clear and are dependent on the performance measures applied.

**Conclusions:**

Sample size recalculation is also applicable in three-stage designs. However, the extent to which recalculation brings benefits depends on which trial characteristics are most important to the applicants.

**Supplementary Information:**

The online version contains supplementary material available at 10.1186/s12874-024-02337-9.

## Introduction

Choosing an adequate sample size is a crucial task when planning a clinical trial. One needs to recruit enough patients to obtain statistically significant evidence for a treatment effect. At the same time, there are multiple reasons why one should not recruit more patients than required: The cost and duration of the trial both grow with the number of patients. Additionally, the number of patients being exposed to trial-related risks should be kept at a minimum. Hence, it is necessary to choose a number of patients, which is neither too large nor too small. The number of patients required to obtain statistically significant evidence for a treatment effect depends on the size of the treatment effect and the endpoint’s variance. Unfortunately, these parameters are unknown when planning a trial. We call this problem in the following the problem of *effect size uncertainty*. The sample size needs to be chosen based on assumed endpoint distribution parameters. If the assumptions are correct, the chosen sample size will be adequate. If the assumptions are wrong, two possible mistakes in sample size planning can be made: First, the sample size could be chosen to low. In this case, the trial has a smaller power then aspired, which is called *underpowered* in the following. This mistake occurs when the assumed treatment effect is larger than the actual treatment effect or when the assumed variance is lower than the actual endpoint variance. Second, the sample size could be chosen too high. In this case, the power is high, but it would have been possible to achieve sufficient power even with fewer patients. We call such a trial *oversized*. Oversizing happens when the assumed treatment effect is lower than the actual treatment effect or when the assumed variance is larger than the actual variance. The fundamental problem in sample size planning is that due to the problem of effect size uncertainty there is always the risk of underpowering and oversizing.

To deal with the problem of effect size uncertainty, two methods are developed, which are to some extent robust against underpowering and oversizing. The first method is sequential testing. Trials with sequential testing unblind the data at different stages of the trial and offer the option to reject the null hypothesis $H_0$ at these stages. Once $H_0$ is rejected, the trial stops. The simplest design with sequential testing is the group sequential design, where the sample sizes of all stages are specified before the beginning of the trial. Such a design provides a remedy to effect size uncertainty, because for large effect sizes $H_0$ likely gets rejected at an early stage, where only a small number of patients has already been recruited. For small effect sizes, the design still offers the option to reject $H_0$ at a later stage, thereby offering the opportunity to recruit enough patients to achieve a high power. A common approach to deal with effect size uncertainty is to specify the sample sizes of a group sequential design such that a targeted power would be achieved for the smallest clinically relevant effect size [1]. The second method providing robustness to effect size uncertainty is sample size recalculation (cf. e.g. [2] for an overview). It extends the method of group sequential designs in so far that the stage-wise sample sizes do not need to be specified before the trial but can be determined based on interim results from the previous stages or other studies that were published in the meantime. In this way, effect estimates from previous stages or other recent trials can be obtained, and based on these effect estimates sample sizes for the remainder of the trial can be determined. Note that unblinded (adaptive) group sequential trial designs naturally come with the shortcoming of unblinding. However, they still find their frequent application as they can be very appealing when having a large insecurity about the underlying parameter values or a high interest in shortening the trial duration. Here, it is important that as few people as possible are unblinded and that the procedure for the sample size update is only available for the statistician responsible for the interim analysis. This allows for fewer conclusions about the effect observed at interim.

One topic of high interest is the evaluation of sample size recalculation rules. There are two perspectives mentioned in the literature in the literature. The *conditional perspective* deems a recalculation rule good, if it ensures stable and high values of the *conditional power* (rejection probability conditional on the interim result) as well as no clear oversizing, despite the problem of effect size uncertainty [3, 4]. An evaluation criterion following the conditional perspective is the *conditional performance score* proposed by Herrmann et al. [3]. In contrast, the *global perspective* rather measures the benefit of sample size recalculation in terms of *global power* (rejection probability before the beginning of the trial) and sample size. According to the global perspective, a recalculation rule should ensure a certain robustness against underpowering (in terms of the global power) and oversizing with regard to the effect size uncertainty [5].

Hence, there are two different perspectives on evaluating sample size recalculation rules. Moreover, there are also different approaches to defining recalculation rules, all of which are plausible in their own way. The *observed conditional power approach* is motivated by the conditional perspective on recalculation rules. This recalculation approach uses interim results of a trial to estimate the treatment effect and to choose then a sample size for the remainder of the trial which guarantees a certain targeted conditional power (e.g. 80% or 90%). Another approach motivated from the conditional perspective is the promising zone approach, which works according to a similar principle as the observed conditional power approach but has some additional case distinctions for the choice of the sample size [6]. There are various studies in recent years proposing recalculation rules which yield optimal performance regarding the respectively applied global performance measures [7–10]. Currently, there is no agreement which is the most favorable recalculation rule to apply.

In this work, we do not aim to provide a solution to the choice of the most favorable sample size recalculation rule. Instead, we aim to fill another gap in the literature about sample size recalculation: With very few exceptions [1, 11] the literature on recalculation only focuses on the case of two-stage trials. However, clinical trial designs do not need to be restricted to two stages. Three-stage trials can offer benefits in terms of expected sample size compared to two- or one-stage trials [1] and can add even further flexibility than two-stage designs. The extent of the benefit, however, depends on the explicit trial designs, i.e., the time interval between the final patient (of a stage) reaching the final visit and the decision to stop, where patients are still enrolled in the trial. As the problem of effect size uncertainty is also relevant for three-stage trials, it is worthwhile to examine the potential benefits of recalculation for these designs. In this paper, we apply concepts from recent research on recalculation in two-stage trials to the case of recalculation in three-stage trials. In detail, this paper offers the following new contributions to the literature: We show how conditional and global performance measures can be applied to the case of recalculation in three-stage trials. Regarding the conditional performance measures, we extend the conditional performance score [3] to the case of three-stage trials with sample size recalculation. Regarding the global performance measures, we apply a performance measure which calculates a trade-off between (global) power and sample size and which is inspired by the approach by Jennison & Turnbull [7]. Having developed appropriate performance measures, we then demonstrate how recalculation rules can be extended to the case of three-stage trials. We demonstrate the application of the performance measures and the respective recalculation rules in a simulation study. Given the empirical results, we assess the potential benefits of applying a three-stage design instead of a two-stage design and of applying recalculation instead of a simple group sequential approach. In the discussion of our study, we elaborate on the different options of recalculation in three stage trials.

## Notation and setting

### Three-stage trials

In this paper, we consider the case of comparing an intervention group (I) with a control group (C), with endpoint distributions given by

$$\begin{aligned} X_{I}\sim N(\mu _{I},\sigma ^2), X_{C}\sim N(\mu _{C},\sigma ^2). \end{aligned}$$

This means, we assume normally distributed endpoints with a common variance. We do not assume that the variance is known.

To test the alternative hypothesis $H_1:\mu _{I}>\mu _{C}$ against the null hypothesis $H_0:\mu _{I}\le \mu _{C}$, we apply a two-sample t-test statistic, defined by

$$\begin{aligned} Z=\frac{\bar{X}_{I}-\bar{X}_{C}}{\sqrt{\frac{2}{n}}\hat{\sigma }}, \end{aligned}$$

where $\bar{X}_{J}$, with $J=I,C$, denotes a sample average, $\hat{\sigma }$ denotes an empirical estimate of the standard deviation in each group and *n* denotes the per-group sample size. In this paper, we consider the case of a three-stage trial. A detailed description of the theory behind multi-stage trials can be found in the book by Wassmer & Brannath [12]. In this book, it is shown that the following sequential testing method maintains the type I error rate.

We apply the t-test statistic at each stage. Let, $n_1,n_2,n_3$ denote the respective sample sizes per group and stage. For simplicity, we consider the case of equal sample sizes in the intervention and control group. The resulting test statistics $Z_{1},Z_{2},Z_{3}$ are independent and we assume large enough sample sizes, such that they asymptotically follow the distributions

$$\begin{aligned} Z_{i}\sim N\left( \sqrt{\frac{n_i}{2}}\delta ,1\right) \text { with } i\in \{1,2,3\}. \end{aligned}$$

Note that the test statistic distribution only depends on the endpoint distribution via the standardized treatment effect

$$\begin{aligned} \delta :=\frac{\mu _{I}-\mu _{C}}{\sigma }. \end{aligned}$$

At each stage *i*, a combination $Z^{*}_i$ of these test statistics is applied for decision making. The respective test statistics are given by the inverse normal combination test [13]

$$\begin{aligned} Z^{*}_1 & =Z_1,\\ Z^{*}_2 & =\frac{w_{1}\cdot Z_{1}+w_{2}\cdot Z_{2}}{\sqrt{w_{1}^2+w_{2}^2}},\\ Z^{*}_3 & =w_1\cdot Z_{1}+w_2\cdot Z_{2}+w_3\cdot Z_{3}. \end{aligned}$$

The weights $w_i$ are defined in advance of the the trial, with the condition $w_1^2+w_2^2+w_3^2=1$. In this paper, we define $w_1=w_2=w_3=\frac{1}{\sqrt{3}}$ throughout. We choose Pocock’s critical values [14] for early rejection of $H_0$, which we denote by $c_1,c_2,c_3$ and futility stopping with $f_1=f_2=0$, i.e. when the effect points in the wrong direction at one of the first two stages the trial stops with acceptance of $H_0$.

### Analysis of interim results

In three-stage trials, interim results are potentially examined at two time points: after having observed the outcome of the first $n_1$ patients per group, and after having observed the following $n_2$ patients per group.

Having observed the interim results, one can estimate “how far” the trial is from proving the treatment effect at the next interim analysis. More formally, this can be expressed by the probability to reject $H_{0}$, given the observed interim results. This probability is called the *conditional rejection probability* and will be denoted by

$$\begin{aligned} CRP_{\delta }^{(i+1)}(z^{*}_{i},n_{i+1}):=P_{\delta }[Z^{*}_{i+1}>c_{i+1}|Z^{*}_{i}], \end{aligned}$$

where $i\in \{1,2\}$ denotes the first or second interim analysis. Given the distribution of the stage-wise test statistic, we can derive the following equations for the stage-wise conditional rejection probability. The equation for the conditional rejection probability at stage two is given by

1 $$\begin{aligned} CRP_{\delta }^{(2)}(z^{*}_{1},n_{2})=1-\Phi \left( \frac{\sqrt{w_1^2+w_2^2}\cdot c_2-w_1\cdot z^{*}_{1}}{w_2}-\delta \cdot \sqrt{\frac{n_2}{2}}\right) , \end{aligned}$$

where $\Phi$ is the cumulative distribution function of the standard normal distribution. The equation for the conditional rejection probability at stage three is given by

2 $$\begin{aligned} CRP_{\delta }^{(3)}(z^{*}_{2},n_{3})=1-\Phi \left( \frac{c_3-\sqrt{w_1^2+w_2^2}\cdot z^{*}_{2}}{w_3}-\delta \cdot \sqrt{\frac{n_3}{2}}\right) . \end{aligned}$$

For recalculation at the first interim analysis, the probability to reject $H_0$ in the remainder of the trial is important. This probability is called *conditional power* (CP) and consists of the conditional rejection probability at the second and third interim analysis in the following way:

3 $$\begin{aligned} CP_{\delta }(z_1^*,n_2,n_3)= & CRP_{\delta }^{(2)}(z_1^*,n_2)\nonumber \\ & +\int _{f_2}^{c_2}CRP_{\delta }^{(3)}(z_2^*,n_3)f_{Z_2^*|Z_1^*=z_1^*,N_2=n_2,\Delta =\delta }(z_2^*)dz_2^*. \end{aligned}$$

Thereby, $f_{Z_2^*|Z_1^*=z_1^*,N_2=n_2,\Delta =\delta }$ is the conditional density of $Z_2^*$, given first stage test statistic value $z_1^*$, second stage per-group sample size $n_2$, and effect size $\delta$. In this notation, $\Delta$ is a random variable for the effect size, which takes the concrete realization $\delta$.

The concept of the conditional power can be used to decide upon the number of patients to recruit after the interim analysis. This number of patients can be chosen such that the conditional power reaches a certain value. Note, however, that the effect size $\delta$ in the definition of the conditional power is unknown in practice. This is why recalculation rules based on the conditional power need to take uncertainty in the effect size $\delta$ into account.

### Group sequential designs and designs with sample size recalculation

In this study, we examine two kinds of designs: group sequential designs and designs with sample size recalculation. For group sequential designs, the per-group sample sizes of all three stages $n_1,n_2,n_3$ are fixed before the beginning of the trial. For designs with recalculation, the per-group sample sizes $n_2,n_3$ of the stages two and three can be determined during the trial, based on the interim results.

In a three-stage trial, recalculation could take place at the first or at the second interim analysis. In this paper, we only consider the case of recalculation at the first interim analysis in detail and leave the case of recalculation at the second interim analysis for the Discussion part as it is very similar to the well known two-stage adaptive trial with sample size recalculation. When recalculation at the first interim analysis takes place, a number of $n_1$ patients per group has already been recruited and the value $z_1^*$ of the first stage test statistic has been obtained. If the first stage test statistic lies within the continuation region $z_1^*\in [f_1,c_1]$, the trial will go into the second-stage. At this point, recalculation allows us to determine the second-stage and third-stage sample sizes per group $n_2,n_3$. In line with Uesaka et al. [11], we highlight the similarity to the sample size calculation for a common two-stage trial: The sample sizes for the following two stages need to be determined, such that $H_0$ can get rejected with sufficient probability. Just like for a common two-stage trial, the interim results included in $z_1^*$ are available and should provide information about the true standardized treatment effect. This should help to decide about an adequate sample size for the remainder of the trial.

Sample size recalculation at the first interim analysis in a three-stage trial differs from sample size recalculation in a two-stage trial in terms of the remaining sample size of the trial and in terms of conditional power: For recalculation in a two-stage trial, the remaining sample size of the trial is determined by $n_2$. In contrast, for recalculation in a three-stage trial, there is still a second interim analysis in which the trial could either stop for efficacy/futility or continue in the third stage. So, even though the second stage sample size $n_2$ and the third stage sample size $n_3$ (which is only applied if the trial continues in the third stage) has been determined at the first interim analysis, the remaining sample size remains stochastic at this point and is expressed by the term $n_2+n_3\cdot I_{z_2^*\in [f_2,c_2]}$. Similarly, the conditional power in a two-stage trial is simply given by $CP_{\Delta }(z_1^*,n_2)=CRP_{\Delta }^{(2)}(z_1^*,n_2)$, while the Formula (3) for the three-stage trial includes the conditional rejection probability at the third stage and the distribution of the second stage test statistic. It are precisely these differences in terms of remaining trial sample size and conditional power which need to be taken into account when transferring concepts from recalculation rules in two-stage trials to recalculation in three-stage trials.

In principle, it would be possible to choose unequal per-group sample sizes $n_2\ne n_3$ for stages two and three in the same way as it is possible to choose unequal stage-wise sample sizes in a two-stage trial. To keep a clear scope of the research, we restrict our analysis to the case of equal per-group stage-wise sample size $n_2=n_3$ and treat the case of unequal stage-wise sample sizes in the Discussion part.

Note that the choice of the first-stage sample size $n_1$ affects both the benefits of the sequential testing procedure and the potential benefits of recalculation: For recalculation, on the one hand, a small first-stage sample size yielding little information about the underlying effect size can only be of limited value for the choice of the remaining sample size. On the other hand, a high first-stage sample size means that a large share of the required patients has already been recruited and the impact of recalculation to adjust the number of further recruitments is limited. The sequential testing procedure is also affected by the choice of $n_1$, as a low choice of $n_1$ reduces the probability to stop for efficacy at the first interim analysis and a high choice of $n_1$ makes an efficacy stop at the first interim analysis likely but reduces the benefits of sample size reduction if $n_1$ is already large. There is no consensus about the ideal choice of sample sizes for an interim analysis. In the two-stage recalculation literature, there exist approaches for optimizing the choice of $n_1$ according to the criteria, the applying statistician deems most important [8]. For three-stage trials, such approaches are so far missing. In our paper, we apply a group sequential design with equal sample sizes per stage, where the sample sizes are chosen in order to reach a pre-specified power $1-\beta$ at an assumed effect size. The considered designs with recalculation use the same first-stage sample size but allow for a flexible sample size in the remainder of the trial.

## Evaluation of recalculation rules

A recalculation rule is intended as a remedy for the problem of uncertainty about the standardized treatment effect. Ideally, the design with recalculation should perform well over a range of standardized treatment effects $\delta$ of interest. Which values of $\delta$ are of interest should thereby be decided based on considerations about the minimally clinically relevant effect and/or logistical restrictions in terms of sample sizes, a maximum plausible effect, and assumptions about the endpoint standard deviation. It is a common approach in the literature to evaluate designs with recalculation over an interval of values for the (standardized) treatment effect [1, 7, 15, 16]. In the following, we provide performance measures $S(\delta )$, measuring how well the sample size is chosen if the underlying standardized treatment effect is $\delta$. We will then examine this performance over a range of effect sizes $\delta$. Ideally, a design with recalculation should provide good performance over the whole range of effect sizes considered.

With regard to the concepts of oversizing and underpowering, we consider a performance as good if the design achieves a high power at $\delta$ while at the same time not requiring too many patients. What a “high” power is can be measured in comparison with a certain target power $1-\beta$ (80% or 90% are usually targeted in practice). If the power falls below $1-\beta$, this should be indicated by a worse performance score. What “too many” patients are can be measured in comparison to the number of patients necessary to achieve a power of $1-\beta$ at a standardized treatment effect $\delta$. If the design chooses a sample size which is larger than necessary to achieve a power of $1-\beta$, this should also be punished by the performance measure.

While the basic idea of how a performance measure *S* should work is clear (quantifying underpowering/oversizing over a range of $\delta$), the literature does not agree on some aspects of the evaluation. In particular, there are two perspectives on evaluation of recalculation rules: the *global perspective* [5] and the *conditional perspective* [3]. The global perspective evaluates a design at each $\delta$ with regard to the (global) power and sample size. This can be interpreted as an assessment based on the information level before the beginning of the trial: I.e. there is an initial assumption of a clinically relevant effect size and the range in which the true effect size is likely to lie. There is agreement, on how much sample size would be acceptable to use. And there are not yet any interim results. Given this level of information, the global measures indicate whether a design can achieve the required power over the range of plausible and relevant effect sizes while complying with sample size restrictions. The conditional perspective works slightly differently: it assumes that in the situation where the recalculation is performed, the interest lies in the conditional power rather than the global power (because the level of information changed, e.g. with interim results being available). Accordingly, it evaluates the designs with regard to conditional power and sample size. As both evaluation perspectives have their advantages, we apply both evaluation principles in the following.

### Global performance perspective

The most commonly applied global performance criteria for recalculation rules are the (global) power $Pow_{\delta }$ and expected sample size $E_{\delta }[N]$. In line with various studies in the literature on recalculation [1, 15, 16], we report these global performance criteria in our simulation study over a range of effect sizes $\delta$. Examining power and expected sample size is very helpful when evaluating the global performance. However, it is hard to derive how good or bad a design is when examining these criteria individually: An oversized design performs bad in terms of expected sample size, but most likely good in terms of power. However, is its performance better or worse than a design with lower sample size which is slightly underpowered?

**Fig. 1:**
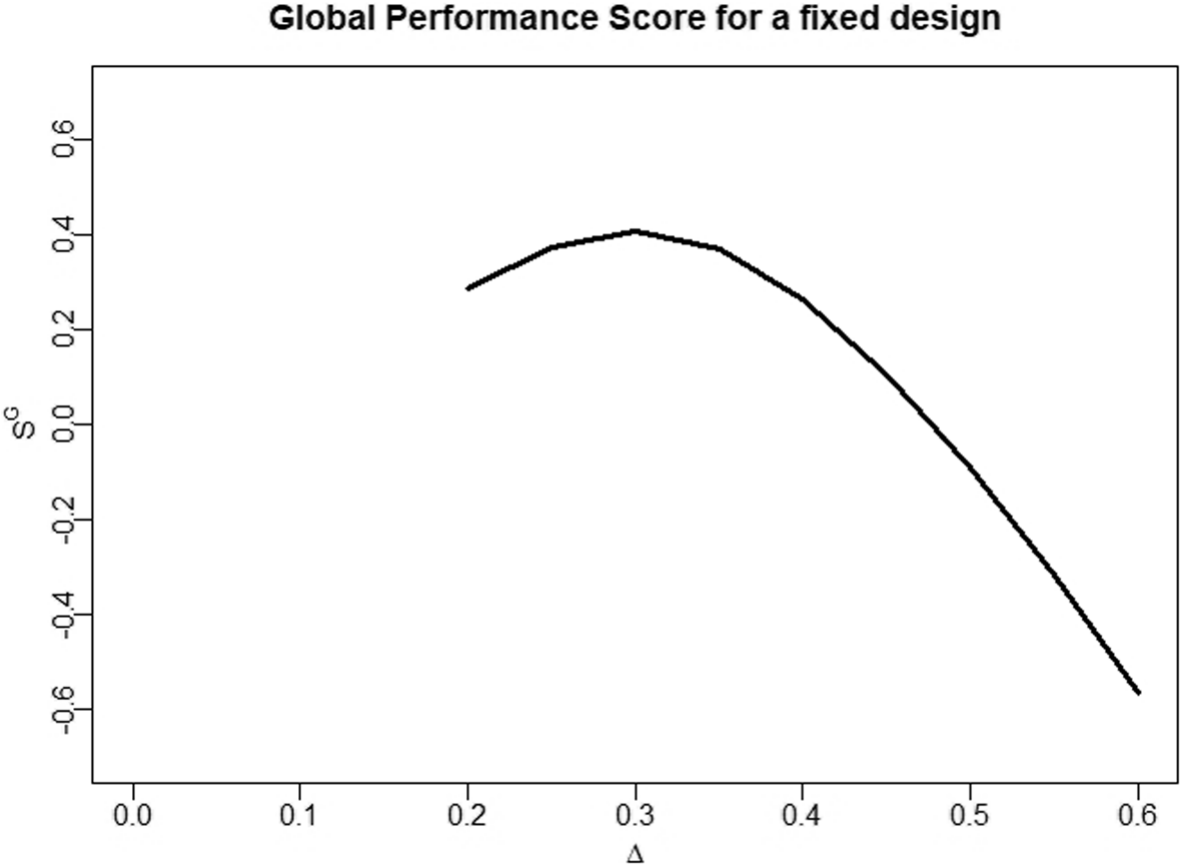
Global performance score $S^G$ for a fixed design, having power $1-\beta =0.8$ at effect size $\delta =0.3$. Smaller effect sizes than 0.2 are deemed clinically irrelevant or unfeasible in terms of the required sample size and therefore performance is not evaluated there

Instead of examining power and sample size individually, it can be helpful to examine them in a combined performance score. Such a performance score can express the tradeoff

$$\begin{aligned} S^{G}(\delta )=Pow_{\delta }-\gamma _{\delta }\cdot E_{\delta }[N] \end{aligned}$$

between power and expected sample size. Optimization of global scores involving a linear tradeoff between power and sample size has already been studied by Jennison & Turnbull [7] and Kunzmann & Kieser [9]. In this research, we apply the tradeoff value

$$\begin{aligned} \gamma _{\delta }:=\frac{\partial Pow_{\delta }(N)}{\partial N}|_{N=N_{fix}(\delta )}, \end{aligned}$$

where $N_{fix}(\delta )$ is the fixed design sample size required to obtain a power of $1-\beta$ at effect size $\delta$. In this way, the ideal score values at each $\delta$ are achieved when the power is close to $1-\beta$ and the expected sample size is close to $N_{fix}(\delta )$. We say “close to”, because multi-stage designs achieve the same power as fixed designs while having lower expected sample sizes. This leads to an ideal trade-off $S^{G}(\delta )$ where the power is slightly above $1-\beta$ and the expected sample size is slightly below $N_{fix}(\delta )$. For fixed designs, the optimal tradeoff would be exactly at $1-\beta$ and $N_{fix}(\delta )$. To illustrate the idea of the score, Fig. 1 shows the performance of a fixed design regarding $S^G$.

The score $S^G$ provides a good summary measure of power and expected sample size. For effect sizes where the designs are underpowering or oversizing, the values of $S^G$ will drop. Another reason why we decided to apply this global performance score is that it is relatively simple to derive recalculation rules maximizing this score (as previously similarly done in e.g. [17, 18]). Evaluating such score-optimized recalculation rules can help us to judge the potential of recalculation rules to prevent underpowering and oversizing. If the optimal recalculation rule does not increase $S^G$ notably compared to a group sequential design, the potential of recalculation, considered from a global perspective, is limited.

Global performance measures, like the global power, the expected sample size, and the $S^G$ score are highly useful criteria given the information level before the beginning of the trial (i.e. no interim results observed yet, some initial assumptions about the effect size). Given this level of information, the global performance measures then indicate whether a design ensures enough power over the range of plausible effect sizes and how much sample size it requires. Once the trial has started, some of these initial assumptions might change: even smaller effect sizes might become relevant (because a competing drug showed unexpected side effects) or the institution conducting a trial is willing and able to recruit more/less patients than initially deemed feasible. In addition, interim results become available at the first interim analysis. So, conditional performance measures gain importance at this point.

### Conditional performance perspective

The conditional performance score $S^{C}$ allows a comparison of different sample size recalculation rules when $z_1 \in [f_1, c_1]$ [3]. The basic idea is to evaluate both the (observed) conditional power and total recalculated per-group sample size regarding their location (*l*) and variation (*v*). This leads to four components (here presented with an equal weighting), which together build a score of the form

4 $$\begin{aligned} S^{C}(\delta ) = \frac{1}{4} \cdot l_{CP}(\delta ) + \frac{1}{4} \cdot v_{CP}(\delta ) + \frac{1}{4} \cdot l_{N}(\delta ) + \frac{1}{4} \cdot v_{N}(\delta ). \end{aligned}$$

Here $l_{CP}$ and $v_{CP}$ are the location and variation components of the (observed) conditional power, while $l_{N}$ and $v_{N}$ denote the location and variation component of the sample size. The four components and thereby the whole score can take values between 0 and 1, where 1 refers to the ideal performance and 0 to the worst possible performance.

In the following, we describe the definition of the location and variation components in more detail. The location components measure the difference between the expected value of (observed) conditional power respectively sample size from a corresponding target value. This difference is then scaled by dividing by the maximal possible difference. The location component of the (observed) conditional power is given by

$$\begin{aligned} l_{CP}(\delta ) = 1-\frac{|E[CP_{\hat{\delta }}(Z_1, N_{rec})]-CP_{target, \delta }|}{1-\alpha }. \end{aligned}$$

Thereby, $CP_{target, \delta }$ denotes the target value for the observed conditional power. It is defined depending on the effect size: If $\delta$ is large enough to reach a power of $1-\beta$ with a sample size smaller or equal to the maximum allowed sample size $n_{max}$, the target value is $1-\beta$. If the effect size is too small, the target value is $\alpha$. The location component of the sample size is defined as

$$\begin{aligned} l_{N}(\delta ) = 1-\frac{|E[N]-N_{target, \delta }|}{n_{max}-n_{1}}. \end{aligned}$$

In this equation, $N_{target,\delta }$ denotes the target value for the sample size. If the effect size is large enough such that $n_{fix}(\delta )$ is smaller than $n_{max}$, the target value is $n_{fix}(\delta )$. If the effect size is too small, the target value is $n_1$.

The variation components of the score measure the variance of the observed conditional power and sample size, standardized by the maximum possible variance. They are defined by

$$\begin{aligned} v_{CP}(\delta ) = 1- \sqrt{\frac{Var(CP_{\hat{\delta }}(Z_1, N_{rec}))}{1/4}}, \end{aligned}$$

and

$$\begin{aligned} v_{N}(\delta ) = 1- \sqrt{\frac{Var(N)}{{\left( (n_{max}-n_1)/2 \right) }^2}}. \end{aligned}$$

Thereby, the maximum possible variances are 1/4 and ${\left( (n_{max}-n_1)/2 \right) }^2$, respectively.

Initially, the conditional performance score was defined for sample size recalculation in two-stage trials [3]. However, the definition of the conditional performance score can also be applied to recalculation at the second stage of three-stage trials. There are only minor changes necessary: A recalculation rule at the first interim analysis chooses the sample sizes $n_2$ and $n_3$. Hence, the total sample size to evaluate becomes $N=n_1+n_2+n_3$. The effect of the choice of $n_2$ and $n_3$ on the observed conditional power can be calculated, using Eq. (3), as

$$\begin{aligned} CP_{\hat{\delta }}(z_1^*,n_2,n_3)= & CRP_{\hat{\delta }}^{(2)}(z_1^*,n_2)\\ & +\int _{f_2}^{c_2}CRP_{\hat{\delta }}^{(3)}(z_2^*,n_3)f_{Z_2^*|Z_1^*=z_1^*,N_2=n_2,\hat{\delta }}(z_2^*)dz_2^*. \end{aligned}$$

Apart from these slight modifications, the conditional performance score can be applied to three-stage trials in the same way as to two-stage trials.

## Recalculation

In this paper, we consider the principle of sample size recalculation, based on an unblinded interim analysis. There is vast literature about such recalculation procedures in two-stage trials [6, 7, 10, 15]. Such trials start with recruiting a number $n_1$ patients per group, which is fixed before the beginning of the trial. At an interim analysis based on these patients, the value $z_1^*$ of the first stage test statistic is then calculated. The second stage per-group sample size $n_2$ is then calculated based on $z_1^*$. To represent this functional relationship, we apply the notation

$$\begin{aligned} n_2=n_2(z_1^*). \end{aligned}$$

For three-stage trials, there is the possibility to recalculate at the first and second interim analysis. We consider recalculation at the first interim analysis. Hence, not only the second stage per-group sample size $n_2$, but also the third stage per-group sample size $n_3$ can be determined at this point. So, recalculation rules from two-stage trials need to be modified in that they yield suitable sample sizes $n_2=n_2(z_1^*)$ and $n_3=n_3(z_1^*)$, based on the interim result $z_1^*$.

For practical reasons, it is plausible to assume that the recalculated sample size could not be chosen arbitrarily large. For this reason, we specified a certain maximum sample size $n_{max}$ for the complete trial. All of the described recalculation rules may yield a number between 0 and $n_{max}-n_1$ patients for the following two stages of the trial.

### Sample size-optimized recalculation

Jennison & Turnbull [7] provide a recalculation rule for two-stage trials, which solves the following constrained optimization problem: minimize $E_{\delta }[N]$ under the constraint $Pow_{\delta }\ge 1-\beta$, for a given effect size $\delta$. They show that the solution to this optimization problem maximizes the performance criterion

$$\begin{aligned} Pow_{\delta }-\gamma \cdot E_{\delta }[N] \end{aligned}$$

for a certain constant $\gamma$. The solution is given by

$$\begin{aligned} n_2(z_1^*)=argmax_{n}\left( CP_{\delta }(z_1^*,n)-\gamma \cdot n\right) . \end{aligned}$$

For each $\gamma$, a recalculation rule can be derived from the above equation. The smaller $\gamma$ is chosen, the higher the power $Pow_{\delta }$ will be. To solve the constrained optimization problem minimizing $E_{\delta }[N]$ under the constraint $Pow_{\delta }\ge 1-\beta$, one only needs to systematically try different values of $\gamma$, until one has the $\gamma$ which yields a recalculation rule for which $Pow_{\delta }= 1-\beta$. This recalculation rule will necessarily solve the constrained optimization problem.

Jennison & Turnbull [7] also explained how to extend this principle to finding a recalculation rule, which minimizes the expected sample size, taking into account uncertainty in the effect size represented by a prior $f_{\Delta }$. The resulting optimization criterion is

5 $$\begin{aligned} \int \left( Pow_{\delta }-\gamma \cdot E_{\delta }[N]\right) f_{\Delta }(\delta )d\delta \end{aligned}$$

and the respective recalculation rule fulfills

$$\begin{aligned} n_2(z_1^*)=argmax_{n}\left( \int CP_{\delta }(z_1^*,n)f_{\Delta |Z_{1}^*=z_{1}^*}(\delta )d\delta -\gamma \cdot n\right) , \end{aligned}$$

where $f_{\Delta |Z_{1}^*=z_{1}^*}(\delta )$ is the posterior density for the effect size. In the same way as for a fixed effect size, systematic trial of different values for $\gamma$ yields a solution to a constrained optimization problem: Minimize the expected sample size $\int E_{\delta }[N]f_{\Delta }(\delta )d\delta$ under the constraint $\int Pow_{\delta }f_{\Delta }(\delta )d\delta \ge 1-\beta$ for the expected power.

In this work, we extend the approach by Jennison & Turnbull [7] to the case of recalculation at the first interim analysis of a three-stage trial. In the Appendix, we derive the sample size-optimized recalculation rule

$$\begin{aligned} \left( n_2(z_1^*),n_3(z_1^*)\right) = & argmax_{n_2,n_3}TO^{(2)}_{z_1^*,\delta }(n_{2})\\ & +\int _{f_2}^{c_2} \left( TO^{(3)}_{z_2^*,\delta }(n_{3})\right) f_{Z_2^*|Z_1^*=z_1^*,N_2=n_2,\Delta =\delta }(z_2^*)dz_2^*\text { with}\\ TO^{(i+1)}_{z_i^*,\delta }(n_{i+1}):= & CRP^{(i+1)}_{\delta }(z_{i}^*,n_{i+1})-\gamma \cdot n_{i+1} \text { for i=1,2}, \end{aligned}$$

for a fixed effect size $\delta$. $TO^{(i+1)}_{z_i^*,\delta }(n_{i+1})$ can be interpreted as a trade-off between the rejection probability and the chosen sample size.

For uncertainty in the effect size, represented by the prior $f_{\Delta }$, we derive the recalculation rule

$$\begin{aligned} \left( n_2(z_1^*),n_3(z_1^*)\right) = & argmax_{n_2,n_3}\int \left( TO^{(2)}_{z_1^*,\delta }(n_{2})+\int _{f_2}^{c_2} \left( TO^{(3)}_{z_2^*,\delta }(n_{3})\right) f_{Z_2^*|Z_1^*=z_1^*,N_2=n_2,\Delta =\delta }(z_2^*)dz_2^*\right) \nonumber \\ & \cdot f_{Z_1^*|\Delta =\delta }(z_1^*)\cdot f_{\Delta }(\delta ). \end{aligned}$$

### *S*^*G*^ score-optimized recalculation

The Jennison & Turnbull [7] approach and our derived modification for the case of three-stage trials yields a recalculation rule, which maximizes the trade-off

$$\begin{aligned} Pow_{\delta }-\gamma \cdot E_{\delta }[N] \end{aligned}$$

for a fixed $\gamma$. A slight modification of the approach leads to an optimization of the global score $S^G$, defined by

$$\begin{aligned} Pow_{\delta }-\gamma _{\delta }\cdot E_{\delta }[N]. \end{aligned}$$

We simply need to apply the effect-dependent value $\gamma _{\delta }:=\frac{\partial Pow_{\delta }(N)}{\partial N}|_{N=N_{fix}(\delta )}$ in the respective equations. Hence, the trade-off functions in the recalculation rule definition of the last section become

$$\begin{aligned} TO^{(i+1)}_{z_i^*,\delta }(n_{i+1})=CRP^{(i+1)}_{\delta }(z_{i}^*,n_{i+1})-\gamma _{\delta }\cdot n_{i+1}\ \text {for}\ i=1,2. \end{aligned}$$

This modified trade-off specification has the effect that the resulting recalculation rules optimize the global score defined in “Global performance perspective” section. The main difference to the Jennison & Turnbull approach is that our performance criterion creates more incentive to maintain a high power at low effect sizes and save sample size at high effect sizes.

In our simulation study, we apply the restriction $n_2=n_3$ when performing recalculation by score-optimization. Note that it is also possible to apply the recalculation rule without the restriction of equal sample sizes for the stages 2 and 3. In this case, one would need, for a given $z_1$, to calculate $\int \left( TO^{(2)}_{z_1^*,\delta }(n_{2})+\int _{f_2}^{c_2} \left( TO^{(3)}_{z_2^*,\delta }(n_{3})\right) f_{Z_2^*|Z_1^*=z_1^*,N_2=n_2,\Delta =\delta }(z_2^*)dz_2^*\right) \cdot f_{Z_1^*|\Delta =\delta }(z_1^*)\cdot f_{\Delta }(\delta )$ for each possible combination of $n_2$ and $n_3$ and then choose the combination of sample sizes, which yields the maximum.

### Observed conditional power approach

An alternative way to choose the recalculated sample size is the observed conditional power approach. This approach uses an effect estimate $\hat{\delta }$ at the first interim analysis and chooses the recalculated per-group sample size $n_2$ such that the observed conditional power reaches a certain target value

$$\begin{aligned} CP_{\hat{\delta }}(z_1^*,n_2,n_3)=1-\beta . \end{aligned}$$

In previous literature, this approach is mostly applied for recalculation in a two-stage trial. An exception is the study by Uesaka et al. [11], where the observed conditional power approach is applied at the first interim analysis of a three-stage trial. We follow their approach in this paper. The sample size for each interim result $z_1^*$ can by obtained by using Eq. (3). We only need to plug-in the effect estimate $\hat{\delta }=\sqrt{\frac{2}{n_1}}z_1^*$ for the true effect size $\Delta$ and then calculate for each possible per-group sample size *n* the conditional power $CP_{\hat{\delta }}(z_1^*,n,n)$. We then choose the smallest per-group sample size *n*, which fulfills $CP_{\hat{\delta }}(z_1^*,n,n)\ge 1-\beta$ or $n=\frac{n_{max}-n_{1}}{2}$. We then set $n_2=n_3=n$. Note that there exists also the option to use unequal sample sizes for the stages 2 and 3.

## Simulation study

In our simulation study, we compared five different designs: A two-stage group sequential design (i.e. with constant sample sizes per stage), a three-stage group sequential design as both described in “Group sequential designs and designs with sample size recalculation” section, a three-stage design with the observed conditional power approach for recalculation, a three-stage design with the expected sample size minimization approach for recalculation by Jennison & Turnbull [7] (see “Sample size-optimized recalculation” section), and a three-stage design with the $S^G$-optimization approach for recalculation (see “SG score-optimized recalculation” section).

We evaluated the performance over the range [0, 0.6] of effect sizes $\delta$. For effect sizes of this magnitude, sufficient sample size is required, such that the application of multi-stage designs in practice is conceivable. The effect size range of [0, 0.6] was also applied in previous studies about sample size recalculation [3, 19]. We powered the group sequential designs for $\delta =0.3$ as the assumed underlying effect size before the start of the trial. Therefore, the quality of a recalculation rule is judged by its ability to avoid underpowering for $\delta <0.3$ and oversizing for $\delta >0.3$. We choose the maximum sample size $n_{max}$ so that a fixed design could achieve a power of $1-\beta =0.8$ with $n_{max}$ patients per group under an effect size of $\delta =0.2$. In this way, the recalculation rules are theoretically able to prevent underpowering for effect sizes $\delta \ge 0.2$. Smaller effect sizes are deemed not feasible in terms of sample sizes and logistics.

The simulations were conducted using R version 4.2.2 [20], and the results for the group sequential designs were obtained using the package rpact [21].

### Simulation settings

For the group sequential designs, we applied Pocock efficacy boundaries [14] and futility stops for interim test statistic results below zero. For both the two-stage and the three-stage designs, we set the first-stage per-group sample size to $n_1=70$. For the two-stage group sequential design, we set the second-stage per-group sample size to 140. For the three-stage group sequential design, we set the second and third-stage per-group sample sizes both to 70. In this way, both the two-stage and the three-stage group sequential design have their first interim analysis based on the results of 70 patients per group and recruit in total 210 patients per group, if the trial enters the respective final stage. With these sample size choices, the group sequential designs both achieve a power of $1-\beta =0.8$ at an effect size of $\delta =0.3$. The fact that the sample size for the first interim analysis is equal for all designs considered is important, because we applied the conditional performance score methodology, which compares designs conditional on the results at interim analysis. For a meaningful comparison, it was necessary that this interim analysis was at the same time for the different designs.

The recalculation rules were specified in the following way: We set the maximum per-group sample size for the whole trial to $n_{max}=393$. Therefore, after the first stage with $n_1=70$ patients per group, the recalculation rules where restricted to recruit between 0 and 323 more patients for the remainder of the trial. For the observed conditional power recalculation approach, we set the targeted conditional power to 0.8. For the sample size optimization approach, we set the cost of sample size to $\gamma =0.0028$. In this way, the design obtains a power of $1-\beta =0.8$ at the effect size $\delta =0.3$. For the optimization of $S^G$, the cost of sample size was set to $\gamma _{\delta }:=\frac{\partial Pow_{\delta }(N)}{\partial N}|_{N=N_{fix}(\delta )}$ for $\delta \in \{0.2, 0.25,0.3, 0.35, 0.4, 0.45, 0.5, 0.55, 0.6\}$. In this way, the score is optimized over the effect sizes $\delta \in [0.2,0.6]$ under consideration.

The target parameters $CP_{target,\delta }$ and $N_{target,\delta }$ in the definition of the conditional performance score are functions depending on the effect size $\delta$. They are defined according to “Conditional performance perspective” section.

### Results with respect to global performance

**Fig. 2:**
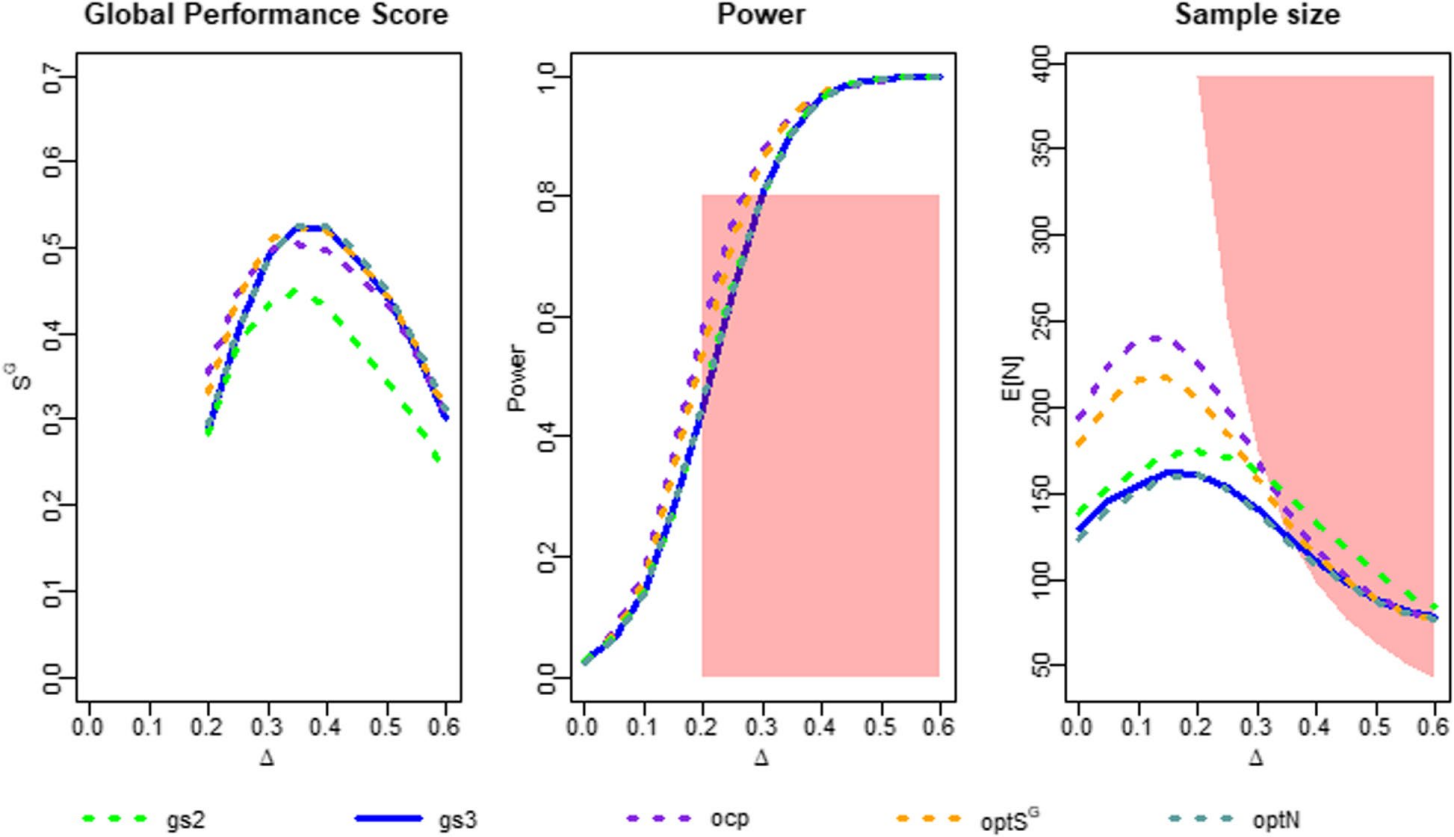
Global performance measures of the different designs. “*gs*2”, “*gs*3” denote the group-sequential designs with 2 or 3 stages. “*ocp*”, “$optS^G$”, “*optN*” denote the three-stage designs with recalculation at the first interim analysis using the observed conditional power approach, the optimization of $S^G$ or the Jennison & Turnbull approach to optimize the expected sample size. The red areas mark regions of underpowering (power of less than $1-\beta$) and oversizing (expected sample size higher than $N_{fix}(\delta )$)

To examine the global performance of our designs, we calculated the power and the expected sample size for all the designs at each effect size $\delta$. For the effect sizes deemed relevant ($\delta \in [0.2,0.6]$), we also calculated the global score $S^G$. The results are illustrated in Fig. 2 and provided in Table 1. Regarding the global score, we can see that for effect sizes of $\delta \ge 0.3$, the two-stage design performs worse than all the three-stage designs. There is a simple explanation for this: The additional interim analysis with the possibility of an efficacy stop reduces the expected sample size compared to the two-stage designs (see plot on the right), while the power curves of these designs are almost identical (see plot in the middle).

**Table 1:** Global performance measures of the simulation. “*gs*2”, “*gs*3” denote the group-sequential designs with 2 or 3 stages. “*ocp*”, “$optS^G$”, “*optN*” denote the three-stage designs with recalculation at the first interim analysis using the observed conditional power approach, the optimization of $S^G$ or the Jennison & Turnbull approach to optimize the expected sample size

		Effect sizes $\delta$						
Design	Perf. measure	0.0	0.1	0.2	0.3	0.4	0.5	0.6
*gs*2	$Pow_{\delta }$	0.027	0.145	0.458	0.814	0.969	0.996	1.000
	$E_{\delta }[N]$	137.4	165.3	175.0	162.9	135.7	106.1	84.8
	$S^G$	...	...	0.283	0.447	0.427	0.333	0.237
*gs*3	$Pow_{\delta }$	0.026	0.149	0.457	0.803	0.964	0.997	1.000
	$E_{\delta }[N]$	128.2	157.4	161.9	140.8	112.0	89.8	77.6
	$S^G$	...	...	0.295	0.487	0.517	0.436	0.302
*ocp*	$Pow_{\delta }$	0.025	0.184	0.582	0.873	0.964	0.992	0.999
	$E_{\delta }[N]$	191.5	243.5	226.7	168.4	119.4	90.6	76.9
	$S^G$	...	...	0.356	0.494	0.487	0.426	0.308
$optS^G$	$Pow_{\delta }$	0.025	0.169	0.540	0.862	0.972	0.996	1.000
	$E_{\delta }[N]$	176.3	219.0	206.8	159.3	115.4	89.3	76.7
	$S^G$	...	...	0.333	0.504	0.511	0.438	0.310
*optN*	$Pow_{\delta }$	0.025	0.146	0.456	0.798	0.957	0.994	1.000
	$E_{\delta }[N]$	122.2	154.6	160.4	139.0	109.9	88.2	76.7
	$S^G$	...	...	0.295	0.486	0.518	0.443	0.310

When examining the global performance score results of the three-stage designs, we note that the group sequential design and the design with sample size-optimized recalculation perform very similar. The slight advantage of the sample size-optimized recalculation rule comes from a reduction of the expected sample size from 115.8 to 114.2 over the range of $\delta$ between 0.2 and 0.6, compared to the group sequential design. The power curves of these two designs are almost identical. In contrast, the observed conditional power approach and the $S^G$-optimized recalculation approach perform notably different from the group sequential design. With power values of 58% (OCP) and 54% ($S^G$-optimized), they suffer notably less from underpowering at effect size $\delta =0.2$ than the group seuquential design which has a power of 46%. This is why they show a better performance regarding $S^G$ for small effect sizes. However, for effect sizes larger than $\delta =0.3$, they suffer more from oversizing than the group sequential trial.

### Results with respect to conditional performance

**Fig. 3:**
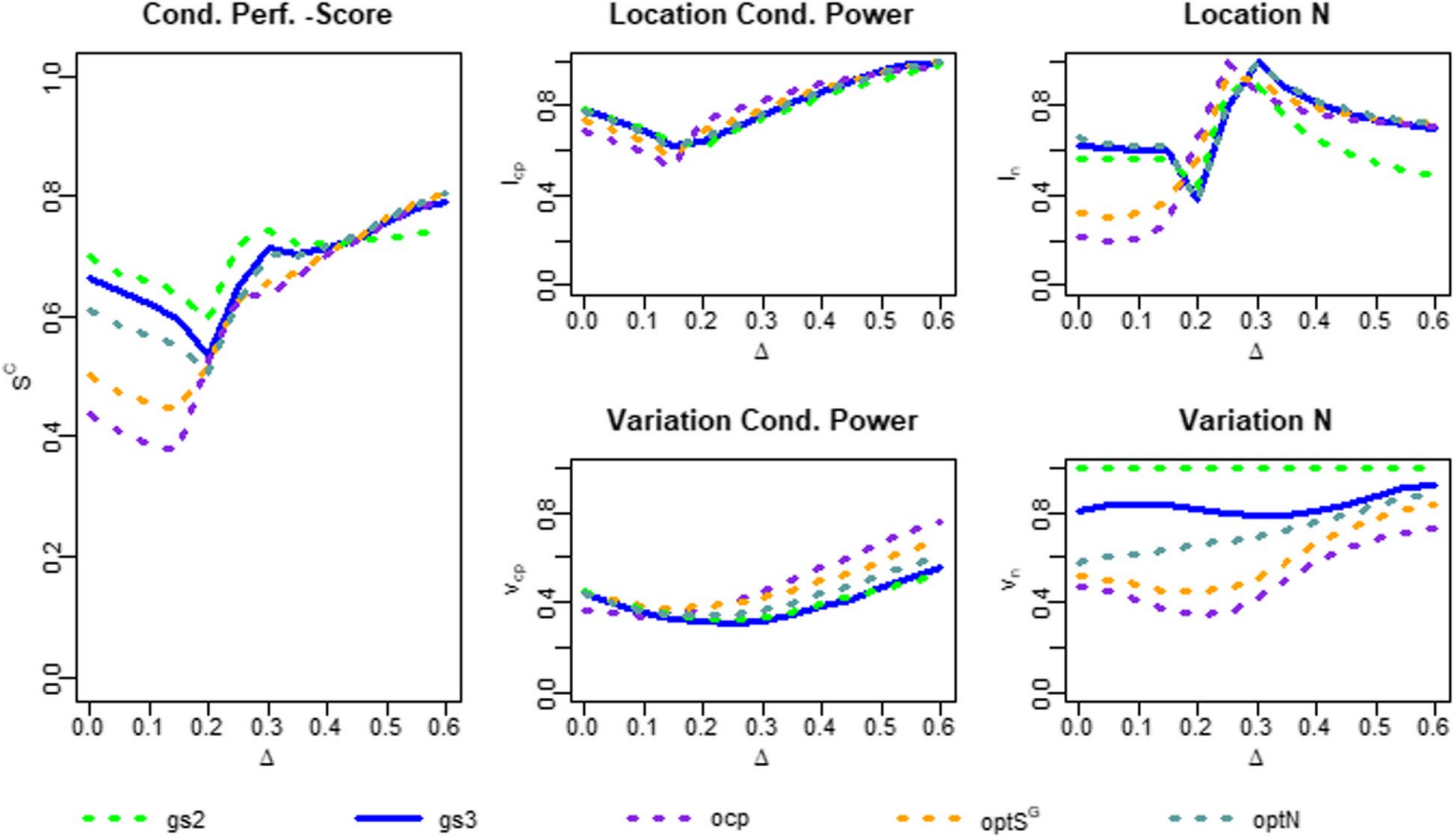
Conditional performance measures of the different designs. “*gs*2”, “*gs*3” denote the group-sequential designs with 2 or 3 stages. “*ocp*”, “$optS^G$”, “*optN*” denote the three-stage designs with recalculation at the first interim analysis using the observed conditional power approach, the optimization of $S^G$ or the Jennison & Turnbull approach to optimize the expected sample size

To evaluate the conditional performance, we calculated the conditional performance score $S^C$ as well as its components $l_{CP},l_{N},v_{CP},v_{N}$ for the location and variation of observed conditional power and sample size. The results are illustrated in Fig. 3 and provided in Table 2. In contrast to the global performance, the two-stage group sequential design performs best according to the conditional performance score for effect sizes lower than 0.4. The reason for this can easily be found when examining the score components: The two-stage group sequential design has no variation in the second-stage sample size (given that the trial continues to the second stage) and hence the component $v_N$ is constantly 1. In contrast, all of the three-stage designs have variation in their sample sizes after the first interim analysis, leading to values of $v_N$ below 1. For effect sizes above 0.4, however, the two stage design is inferior to the three-stage designs. The reason for this is the location of the sample size: Due to its higher expected sample size, the two-stage design suffers more from oversizing then the three-stage designs.

**Table 2:** Conditional performance measures of the simulation. “*gs*2”, “*gs*3” denote the group-sequential designs with 2 or 3 stages. “*ocp*”, “$optS^G$”, “*optN*” denote the three-stage designs with recalculation at the first interim analysis using the observed conditional power approach, the optimization of $S^G$ or the Jennison & Turnbull approach to optimize the expected sample size. $l_{CP}$, $v_{CP}$, $l_{N}$, $v_N$ denote the location and variation components of conditional power and sample size for the conditional performance score $S^C$

		Effect sizes $\delta$						
Design	Perf. measure	0.0	0.1	0.2	0.3	0.4	0.5	0.6
*gs*2	$l_{CP}$	0.789	0.691	0.622	0.739	0.839	0.923	0.992
	$v_{CP}$	0.438	0.365	0.328	0.332	0.385	0.458	0.558
	$l_{N}$	0.567	0.567	0.435	0.890	0.654	0.544	0.485
	$v_{N}$	1	1	1	1	1	1	1
	$S^C$	0.699	0.656	0.596	0.740	0.719	0.731	0.759
*gs*3	$l_{CP}$	0.796	0.689	0.638	0.755	0.868	0.953	0.983
	$v_{CP}$	0.451	0.351	0.311	0.319	0.372	0.448	0.557
	$l_{N}$	0.625	0.606	0.381	0.991	0.811	0.738	0.696
	$v_{N}$	0.808	0.833	0.813	0.784	0.807	0.865	0.934
	$S^C$	0.670	0.620	0.536	0.712	0.715	0.751	0.792
*ocp*	$l_{CP}$	0.694	0.582	0.731	0.819	0.894	0.944	0.981
	$v_{CP}$	0.366	0.345	0.370	0.439	0.544	0.653	0.779
	$l_{N}$	0.218	0.211	0.649	0.864	0.761	0.730	0.717
	$v_{N}$	0.469	0.416	0.337	0.412	0.581	0.682	0.726
	$S^C$	0.437	0.388	0.522	0.634	0.695	0.752	0.801
$optS^G$	$l_{CP}$	0.740	0.634	0.682	0.783	0.871	0.944	0.999
	$v_{CP}$	0.439	0.390	0.378	0.410	0.486	0.573	0.681
	$l_{N}$	0.316	0.323	0.566	0.907	0.788	0.746	0.723
	$v_{N}$	0.517	0.481	0.442	0.504	0.653	0.773	0.842
	$S^C$	0.503	0.457	0.517	0.651	0.699	0.759	0.811
*optN*	$l_{CP}$	0.776	0.670	0.652	0.761	0.858	0.941	0.996
	$v_{CP}$	0.439	0.369	0.338	0.357	0.426	0.512	0.624
	$l_{N}$	0.664	0.615	0.375	0.999	0.824	0.759	0.724
	$v_{N}$	0.570	0.618	0.656	0.688	0.750	0.830	0.893
	$S^C$	0.612	0.568	0.505	0.701	0.714	0.761	0.809

When comparing the three-stage designs with regard to the conditional performance score, the group sequential design performs best, especially for low effect sizes. The reason for this lies in the sample size components: The variation in the sample size is again lower for the group sequential design than for the designs with recalculation. In addition, the $S^G$-optimized and sample size-optimized recalculation rules perform much worse in terms of sample size location for effect sizes $\delta <0.2$. This is because they recruit a large number of patients even though the effect size is too small to achieve sufficient power. A slight benefit of the recalculation rules can be seen in the variation component $v_{CP}$ of the observed conditional power. However, this benefit does not compensate the disadvantage in terms of location and variation of the sample size.

## Discussion

In this paper, we have analyzed sample size recalculation in three-stage clinical trials with a focus on different evaluation perspectives. To this end, we applied sample size recalculation methods from the literature for two-stage clinical trials to the case of recalculation at the first interim analysis in three-stage clinical trials. While an extension of the observed conditional power approach to three-stage trials has already been performed by Uesaka et al. [11], we are, to the best of our knowledge, the first having extended the sample size optimization approach of Jennison & Turnbull [7] to three-stage clinical trials. Apart from an extension of recalculation rules to three-stage trials, we have also extended an evaluation method for recalculation rules, namely the conditional performance score, to the case of three-stage trials.

In terms of global performance, measured by power and expected sample size, the three-stage designs performed notably better than the considered two-stage design. In contrast, the two-stage group sequential design outperformed the three-stage designs with regard to the conditional performance score. This shows that the performance of a recalculation rule strongly depends on the perspective one takes with regard to evaluation. Does the three-stage design’s reduction in expected sample size, from the global perspective outweigh the disadvantage of higher variation of the sample size, which leads to the inferior performance, in terms of the conditional performance score? This question is up to the applicant.We note that the conditional performance score definition can be customized depending on which performance aspects the applicant finds most relevant. In the score definition (4) we applied an equal weighting scheme for the four performance components. However, if an applicant deems expected sample size reduction due to the second interim analysis of a three-stage trial more relevant than the uncertainty in sample size, which it implies, s/he could assign a higher weight to the $l_N$ and a lower weight to the $v_N$ component in the score definition. Such a change in the weighting would lead to a relative performance improvement of three-stage trials compared to two-stage trials, with regard to the conditional performance score.

We also compared global and conditional performance of group sequential three-stage designs and three-stage designs with recalculation. Recalculation leads to a deterioration in terms of conditional performance. This is in line with similar comparisons in the context of two-stage designs [3]. Note that none of the recalculation rules applied in this study were optimized according to the conditional performance score, so there might still be a margin for improvement. However, even for conditional performance score-optimized recalculation rules, the two-stage literature so far only showed limited potential for improvement over group sequential trials [18]. In terms of global performance, the recalculation rules achieved a slight advantage over the group sequential design. This is due to their potential to achieve the same power by a lower expected sample size and by their ability to work against underpowering at a low effect size. The relatively small performance gain compared to group sequential designs is not specific for three-stage trials but was also noted in the two-stage recalculation literature. E.g. Jennison and Turnbull [7] showed that recalculation can only marginally reduce the expected sample size, given power constraints, compared to group sequential designs and Pilz et al. [8] optimized recalculation rules to prevent underpowering at low effect sizes but found that these can lead to very high sample size choices. So, given these results from the two-stage literature, it is not surprising that the simulation did not reveal more significant performance gains of three-stage trials with recalculation.

For any difference between the observed global and conditional performance for the designs, it should be noted that it is not only a matter of the performance perspective (conditional versus global) but also a matter of the definition of the two scores: the conditional performance score includes the variance of the sample size and power while the global score does not. Regarding the robustness against underpowering, it needs to be said that a group sequential design powered for lower effect sizes would also suffer less from underpowering.

In this paper, we restricted our analysis to recalculation at the first interim analysis of a three-stage trial. In this way, the resulting design can be considered as a combination of an adaptive design (with flexible sample size choice at the first interim analysis) and a group sequential design (with the second interim analysis offering the option to stop for efficacy or futility, but not to adapt sample size for the remainder of the trial). So, stages two and three can be interpreted as a common two-stage group sequential design, where the sample size choice has been made in advance (at the first interim analysis). Consequently, stages two and three share typical limitations of group sequential trials. In particular, it is possible that second-stage interim results can suggest that the chosen sample size for the third stage offers low power, and the trial nevertheless continues with the third stage, without an increase in sample size. As there is an ongoing debate about the potential benefits of adaptive designs in terms of flexibility versus the benefits of group sequential designs in terms of simplicity and planning security [7, 10, 22], we deem the suggested three-stage designs of practical relevance, despite their limitations.

An alternative to the suggested design would be to allow recalculation at the second interim analysis instead of at the first interim analysis. Recalculation at the second interim analysis is, in principle, very similar to recalculation in two-stage trials because there is only one remaining stage after the recalculation is performed. Accordingly, recalculation rules from the two-stage trial literature would be applicable without much modification. Hence, an analysis of recalculation at the first interim analysis is arguably of higher research interest. In addition, we are of the opinion that the potential of sample size recalculation is higher at the first interim analysis than at the second interim analysis. This is because the probability of a trial to go into the third stage is lower than the probability to get into the second stage. Hence, a recalculation rule at the third stage is less likely to be applied in the trial. Moreover, at the first interim analysis there is more remaining $\alpha$ to spend than at the second interim analysis (where one additional option for efficacy stop has already passed). Thus, there is a higher potential to affect the power of the design by modifying the sample size at the first interim analysis.

A possible extension of our suggested designs with recalculation at the first interim analysis would be to allow additional recalculation at the second interim analysis. This would sacrifice the advantage of stage two’s and stage three’s group-sequential structure in favor of more flexibility. A proper definition of recalculation rules at the second interim analysis, when the design also allows recalculation at the first interim analysis, is, however, not trivial. This is due to the fact that, in this case, the sampling procedure for the effect estimate at the second interim analysis depends on the results of the first interim analysis [23]. This dependence can make effect estimates biased (which would be problematic for recalculation approaches like OCP, which rely on effect estimates) and generally makes the distribution of such effect estimates or the corresponding second-stage test statistic complex and difficult to express mathematically (which is problematic for optimization approaches like sample size-optimized or $S^G$ score-optimized recalculation). Given this problem of a proper definition of recalculation rules at the second interim analysis, if recalculation at the first interim analysis is allowed, we decided not to include recalculation at the second interim analysis in this study. We encourage further research in this direction, which provides a proper solution for the definition of the recalculation rules at the second interim analysis. Having defined recalculation rules for the second interim analysis, the recalculation rules considered here for the first interim analysis could, with modifications, be applied. In this case, the formulas for the conditional power and expected sample size, on which the observed conditional power, the sample size-optimization, and the $S^G$-optimization approach rely, would need to be modified such that the third-stage per-group sample size $n_3(z_2^*)$ is specified as a function of the second-stage interim result. Apart from this change, the recalculation rules for the first interim analysis can be derived in the same way as we did here.

Another possible extension of the considered methodology would be different types of endpoints. Our paper focuses on continuous endpoints but three-stage trials would also be possible for binary and time-to-event endpoints. Also, the considered recalculation rules could be extended as they rely on the concept of the conditional power, which is also feasible for these other endpoint types. Some aspects of the trial design would become more complicated in the case of time-to-event endpoints: In our paper, we made the standard assumption that the continuous outcome of a patient is observable at the time of recruitment so that the amount of information at the first interim analysis depends directly on the number of recruited patients $n_1$. For time-to-event endpoints, however, the event rate is also important, so that the amount of information not only depends on $n_1$ but also on the event rate. This makes it more complicated to find a right time point for an interim analysis, such that enough information for recalculation is available and at the same time not too many patients have already been recruited. This could also be an interesting aspect for future research.

With this work we shed light on three-stage trials with sample size recalculation and their evaluation. However, in the practical applications, extensive simulation studies are needed for a detailed comparison of realistic options and other sample size recalculation approaches (e.g. [6]).

## Supplementary Information

Supplementary Material 1.

## Data Availability

No original trial data are used in this work. Simulated data and software source code that support the findings of the simulation study can be found in the github repository https://github.com/bokelmab/three_stage_trials.
