# Supplementary material for: Sample size recalculation in three-stage clinical trials and its evaluation
Source: BMC Med Res Methodol. 2024 Sep 25;24:214. doi: 10.1186/s12874-024-02337-9 (PMC11423520; doi:10.1186/s12874-024-02337-9)
Supplement: Supplementary file 1 — Supplementary Material 1. [file 12874_2024_2337_MOESM1_ESM.pdf]

Appendix A: Sample size-optimized recalculation

In the following, we derive recalculation rules at the second stage which optimize a tradeoff between power and expected sample size of the form $Pow_\delta - \gamma \cdot E_\delta[N]$. First, we start by deriving the optimal recalculation rule, for a fixed effect size δ. It holds that

$$
\begin{aligned}
& Pow_\delta - \gamma \cdot E_\delta[N] \\
& = P_\delta[Z_1^* > c_1] + P_\delta[Z_2^* > c_2 \text{ or } Z_3^* > c_3 | z_1^* \in [f_1, c_1], n_2, n_3] - \gamma \cdot n_1 \\
& - \gamma \cdot E_\delta[n_2 + I_{z_2^* \in [f_2, c_2]} \cdot n_3 | z_1^* \in [f_1, c_1], n_2, n_3].
\end{aligned}
$$

Hence, given an interim result $z_1^* \in [f_1, c_1]$, the optimal choices of n_2 and n_3 correspond to a maximization of

$$
\begin{aligned}
& P_\delta[Z_2^* > c_2 \text{ or } Z_3^* > c_3 | z_1^*, n_2, n_3] - \gamma \cdot E_\delta[n_2 + I_{z_2^* \in [f_2, c_2]} \cdot n_3 | z_1^*, n_2, n_3] \\
& = CP_\delta(z_1^*, n_2, n_3) - \gamma \cdot n_2 - \gamma \cdot n_3 \cdot \int_{f_2}^{c_2} f_{Z_2^* | Z_1^* = z_1^*, N_2 = n_2, \Delta = \delta}(z_2^*) dz_2^* \\
& = CRP_\delta^{(2)}(z_1^*, n_2) - \gamma \cdot n_2 + \int_{f_2}^{c_2} \left(CRP_\delta^{(3)}(z_2^*, n_3) - \gamma \cdot n_3 \right) f_{Z_2^* | Z_1^* = z_1^*, N_2 = n_2, \Delta = \delta}(z_2^*) dz_2^*.
\end{aligned}
$$

The pair n_2, n_3 of sample sizes, which are optimal for an interim result z_1^*, needs to fulfill

$$
\begin{aligned}
(n_2(z_1^*), n_3(z_1^*)) = & argmax_{n_2, n_3} TO_{z_1^*, \delta}^{(2)}(n_2) \\
& + \int_{f_2}^{c_2} \left(TO_{z_2^*, \delta}^{(3)}(n_3) \right) f_{Z_2^* | Z_1^* = z_1^*, N_2 = n_2, \Delta = \delta}(z_2^*) dz_2^* \text{ with} \\
TO_{z_i^*, \delta}^{(i+1)}(n_{i+1}) = & CRP_\delta^{(i+1)}(z_i^*, n_{i+1}) - \gamma \cdot n_{i+1} \text{ for } i = 1, 2.
\end{aligned}
$$

Next, we consider the case that there is uncertainty about the effect size, represented by a prior f_Δ. The criterion to maximize at each z_1^* then becomes

$$
\begin{aligned}
&P[Z_2^* > c_2 \text{ or } Z_3^* > c_3 | z_1^*, n_2, n_3] - \gamma \cdot E[n_2 + I_{z_2^* \in [f_2, c_2]} \cdot n_3 | z_1^*, n_2, n_3] \\
&= \int \left(CP_\delta(z_1^*, n_2, n_3) - \gamma \cdot E_\delta[n_2 + I_{z_2^* \in [f_2, c_2]} \cdot n_3 | z_1^*, n_2, n_3] \right) \cdot f_{\Delta | Z_1^* = z_1^*}(\delta) d\delta \\
&= \int \left(TO^{(2)}_{z_1^*, \delta}(n_2) + \int_{f_2}^{c_2} \left(TO^{(3)}_{z_2^*, \delta}(n_3) \right) f_{Z_2^* | Z_1^* = z_1^*, N_2 = n_2, \Delta = \delta}(z_2^*) dz_2^* \right) \cdot f_{\Delta | Z_1^* = z_1^*}(\delta) d\delta.
\end{aligned}
$$

It holds

$$
f_{\Delta | Z_1^* = z_1^*}(\delta) \propto f_{Z_1^* | \Delta = \delta}(z_1^*) \cdot f_\Delta(\delta).
$$

So, the recalculated sample sizes n_2, n_3 can be obtained via the equation

$$
\begin{aligned}
(n_2(z_1^*), n_3(z_1^*)) = argmax_{n_2, n_3} \int \left(TO^{(2)}_{z_1^*, \delta}(n_2) + \int_{f_2}^{c_2} \left(TO^{(3)}_{z_2^*, \delta}(n_3) \right) f_{Z_2^* | Z_1^* = z_1^*, N_2 = n_2, \Delta = \delta}(z_2^*) dz_2^* \right) \\
\cdot f_{Z_1^* | \Delta = \delta}(z_1^*) \cdot f_\Delta(\delta).
\end{aligned}
$$

The sample size-optimized recalculation works by calculating the expression on the right side of the equation for each pair n_2, n_3 and then choosing the pair which maximizes the expression. To do so, we need formulas for the two conditional densities $f_{Z_2^* | Z_1^* = z_1^*, N_2 = n_2, \Delta = \delta}$ and $f_{Z_1^* | \Delta = \delta}$. The latter density is easy to derive, as $Z_1^* |_{\Delta = \delta}$ follows a normal distribution with mean $\sqrt{\frac{n_1}{2}}\delta$ and variance 1. So, it holds

$$
f_{Z_1^* | \Delta = \delta}(z_1^*) = \frac{1}{\sqrt{2\pi}} e^{-\frac{1}{2}\left(z_1^* - \sqrt{\frac{n_1}{2}}\delta\right)^2}.
$$

The conditional density of Z_2^* can be derived from the combined test statistic

$$Z_2^* = \frac{w_1 \cdot Z_1^* + w_2 \cdot Z_2}{\sqrt{w_1^2 + w_2^2}}.$$

From this definition, it follows

$$Z_2^*|_{Z_1^*=z_1^*, N_2=n_2, \Delta=\delta} \sim N\left(\frac{w_1 \cdot z_1^* + w_2 \cdot \sqrt{\frac{n_2}{2}}\delta}{\sqrt{w_1^2 + w_2^2}}, \frac{w_2^2}{w_1^2 + w_2^2}\right)$$

and hence

$$\begin{aligned} f_{Z_2^*|Z_1^*=z_1^*, N_2=n_2, \Delta=\delta}(z_2^*) = \frac{1}{\sqrt{2\pi\sigma^2}} e^{-\frac{1}{2}\left(\frac{z_2^*-\mu}{\sigma}\right)^2} \text{ with} \\ \mu := \frac{w_1 \cdot z_1^* + w_2 \cdot \sqrt{\frac{n_2}{2}}\delta}{\sqrt{w_1^2 + w_2^2}} \\ \sigma^2 := \frac{w_2^2}{w_1^2 + w_2^2}. \end{aligned}$$
